# Probing the Effect of Physiological Concentrations of IL-6 on Insulin Secretion by INS-1 832/3 Insulinoma Cells under Diabetic-Like Conditions

**DOI:** 10.3390/ijms19071924

**Published:** 2018-06-30

**Authors:** Jonathan Barlow, Steven Carter, Thomas P. J. Solomon

**Affiliations:** 1 School of Sport, Exercise and Rehabilitation Sciences, University of Birmingham, Birmingham B15 2TT, UK; SXC752@student.bham.ac.uk (S.C.); t.solomon@bham.ac.uk (T.P.J.S.); 2 Institute for Metabolism and Systems Research (IMSR), University of Birmingham, Birmingham B15 2TT, UK

**Keywords:** pancreatic beta cell dysfunction, insulin secretion, interleukin-6, type 2 diabetes, exercise, skeletal muscle, electrical pulse stimulation, organ crosstalk

## Abstract

Exercise improves insulin secretion by pancreatic beta cells (β-cells) in patients with type 2 diabetes, but molecular mechanisms of this effect are yet to be determined. Given that contracting skeletal muscle causes a spike in circulating interleukin-6 (IL-6) levels during exercise, muscle-derived IL-6 is a possible endocrine signal associated with skeletal muscle to β-cell crosstalk. Evidence to support a role of IL-6 in regulating the health and function of β-cells is currently inconsistent and studies investigating the role of IL-6 on the function of β-cells exposed to type 2 diabetic-like conditions are limited and often confounded by supraphysiological IL-6 concentrations. The purpose of this study is to explore the extent by which an exercise-relevant concentration of IL-6 influences the function of pancreatic β-cells exposed to type 2 diabetic-like conditions. Using insulin-secreting INS-1 832/3 cells as an experimental β-cell model, we show that 1-h IL-6 (10 pg/mL) has no effect on insulin secretion under normal conditions and does not restore the loss of insulin secretion caused by elevated glucose ± palmitate or IL-1β. Moreover, treatment of INS-1 832/3 cells to medium collected from C2C12 myotubes conditioned with electrical pulse stimulation does not alter insulin secretion despite significant increases in IL-6. Since insulin secretory defects caused by diabetic-like conditions are neither improved nor worsened by exposure to physiological IL-6 levels, we conclude that the beneficial effect of exercise on β-cell function is unlikely to be driven by muscle-derived IL-6.

## 1. Introduction

The pathophysiology of type 2 diabetes (T2D) is incompletely understood, but it is generally accepted that hyperglycemia occurs only when insulin resistance is combined with β-cell dysfunction [1]. Defects in β-cell function are therefore essential to the risk and development of T2D [2]. Finding new therapies targeted at improving β-cell function is thus key to restoring normal blood glucose homeostasis in patients with T2D. 

Exercise is central to the primary care for patients with T2D [3] through its action on slowing the progression of glucose intolerance [4]. Glucoregulatory benefits of exercise have been attributed to enhanced insulin sensitivity of peripheral tissues [5] and to improved β-cell function [6,7]. Notably, exercise also restores β-cell mass in rodent models of both T1D [8] and T2D [9]. Biologically active compounds released during exercise likely contribute to the protective effects of exercise in patients with T2D [10,11]. As many of these compounds are released by skeletal muscle [12], it is currently thought that muscle contraction mediates interorgan crosstalk with distant tissues, including β-cells [11]. In this respect, it has been reported that muscle-derived factors (myokines) such as interleukin-6 are accountable for the protective effects of “muscle-conditioned” medium [13] and “exercise serum” [14] against proinflammatory-induced β-cell apoptosis. 

Although exercise-regulated IL-6 is recognised to lower cytokine-induced loss of β-cell mass [13,14], specific effects of IL-6 on β-cell function are less convincing and confusing. For example, IL-6 infusion in mice stimulates [15] and suppresses [16,17] insulin release, while exogenous IL-6 treatment of cultured insulin-secreting cells and isolated islets increases insulin release in some [18,19] but not all studies [20]. A better understanding between the interaction of a transient IL-6 response to acute exercise and its possible effects on β-cell function under diabetic-like conditions is therefore necessary. In this regard we explored if a direct effect of IL-6 at an exercise-relevant concentration mediates insulin secretory function of INS-1 832/3 cells under diabetic-like conditions in vitro. 

## 2. Results

### 2.1. Acute IL-6 Treatment Has No Effect on Glucose-Stimulated Insulin Secretion (GSIS) by INS-1 832/3 Cells at an Exercise-Relevant Concentration

**Figure 1:**
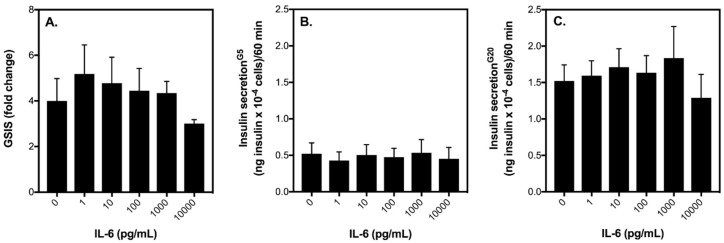
Dose response of IL-6 on glucose-stimulated insulin secretion. INS-1 832/3 cells were grown in fully supplemented RPMI and exposed for 1 h to IL-6 at 0, 1, 10, 100, 1000, or 10,000 pg/mL. The rate of insulin secretion was either normalised to basal insulin release (**A**) or to cell number (**B**,**C**) and was measured at 5 mM glucose (basal) or 20 mM glucose (stimulated). Data are means ± SEM from 5 independent experiments with each condition repeated 4–5 times. Statistical significance of mean differences was tested by one-way ANOVA.

**Figure 2:**
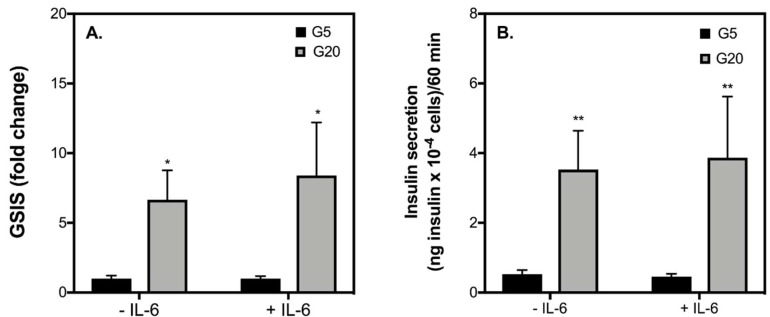
The effect of an exercise-relevant concentration of IL-6 on insulin secretion. INS-1 832/3 cells were grown in fully supplemented RPMI and exposed for 1 h to IL-6 (10 pg/mL). The rate of insulin secretion was either normalized to basal insulin release (**A**) or to cell number (**B**) and was measured at 5 mM glucose (basal—black bars) or 20 mM glucose (stimulated—grey bars). Data are means ± SEM from 4 independent experiments with each condition repeated 4–5 times. Statistical significance of mean differences was tested by 2-way ANOVA: asterisks indicate statistically significant differences from equivalent basal glucose conditions (* *p* < 0.05 and ** *p* < 0.01).

Exposure of INS-1 832/3 cells to increasing concentrations of IL-6 (0–10,000 pg/mL) has no significant effect on glucose-stimulated insulin secretion (GSIS) (Figure 1A). That said, at 10,000 pg/mL, IL-6 tends to lower GSIS, which appears to be a result of suppressed insulin release by INS-1 832/3 cells when stimulated with 20 mM glucose (Figure 1C). Basal insulin secretion is not significantly affected by increasing IL-6 (Figure 1B). Importantly, at a concentration of 10 pg/mL (a concentration that better reflects what is released into circulation following an acute bout of exercise in humans [21]), it appears that IL-6 has no effect on GSIS by INS-1 832/3 cells (Figure 1). To confirm this lack of effect, in a separate set of experiments we incubated healthy INS-1 832/3 cells with 10 pg/mL IL-6 for 1 h and then assessed insulin release at 5 and 20 mM glucose. In healthy INS-1 832/3 cells without IL-6 treatment, insulin secretion is increased to 6-fold in response to 20 mM glucose (Figure 2A), from 0.5 to 3.0 ng insulin × 10^−4^ cells/60 min (Figure 2B). Interestingly, in cells treated with IL-6, insulin secretion is stimulated further by 20 mM glucose to 8-fold (Figure 2A), from 0.5 to 4.0 ng insulin × 10^−4^ cells/60 min (Figure 2B). Importantly, however, this increase in GSIS by cells exposed to IL-6 for 1 h is not statistically significant from control cells (*p* = 0.58).

### 2.2. Acute IL-6 Treatment Neither Worsens nor Improves Insulin Secretory Function by INS-1 832/3 Cells Exposed to Diabetic-Like Conditions

**Figure 3:**
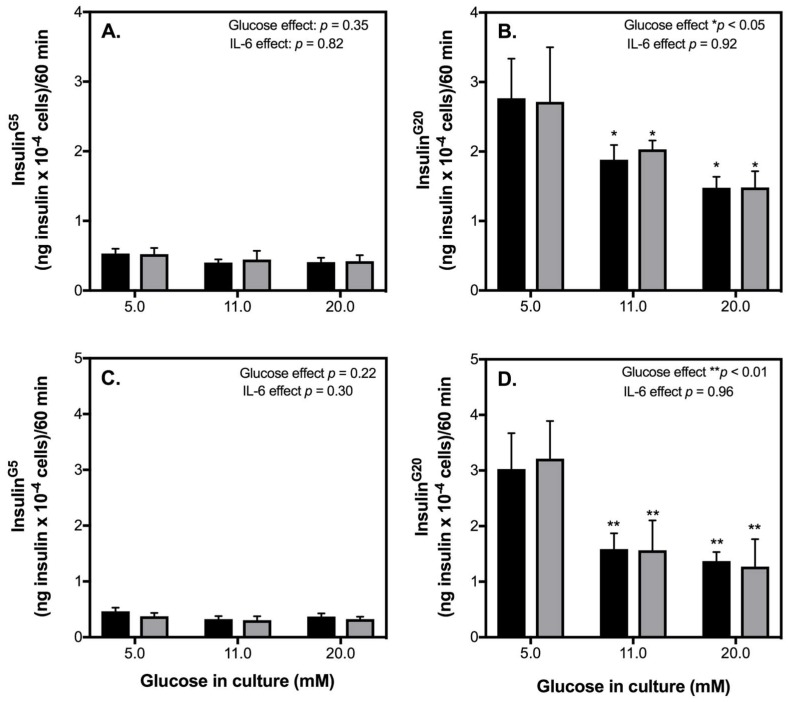
IL-6 does not mediate insulin secretion in glucotoxic or glucolipotoxic cells. INS-1 832/3 cells exposed for 48 h to increasing glucose 5, 11, or 20 mM in RPMI in the presence (**C**,**D**) or absence of BSA-conjugated palmitate (**A**,**B**) were treated with IL-6 (grey bars) or without IL-6 (black bars) for 1 h. IL-6 effects were determined on basal (G5) insulin secretion (**A**,**C**) and high glucose (G20) insulin secretion (**B**,**D**) for glucotoxic (**A**,**B**) and glucolipotoxic (**C**,**D**) cells, respectively. Data are means ± SEM of 4 independent experiments with each condition repeated 4–5 times. Mean differences were tested for statistical significance by 2-way ANOVA: asterisks indicate statistically significant differences from cells cultured in 5 mM glucose (* *p* < 0.05 and ** *p* < 0.01).

Despite the fact that an exercise-relevant concentration of IL-6 has no significant effect in healthy INS-1 832/3 cells, we next explored to what extent IL-6 may alter insulin secretion by INS-1 832/3 cells pre-exposed to glucotoxic or glucolipotoxic diabetic-like conditions. 48-h exposure of INS-1 832/3 cells to increasing glucose without (Figure 3A) or with palmitate (Figure 3C) has no significant effect on basal insulin release and is similar in cells treated with IL-6. On the other hand, 48-h exposure to increasing glucose significantly lowers the amount of insulin secreted by INS-1 832/3 cells in response to 20 mM glucose (Figure 3B). Insulin secretion in response to 20 mM glucose is further attenuated in cells pre-exposed to elevated glucose plus BSA-conjugated palmitate (Figure 3D). This loss of insulin secretory function is unaffected by acute exposure to IL-6 (Figure 3B,D).

**Figure 4:**
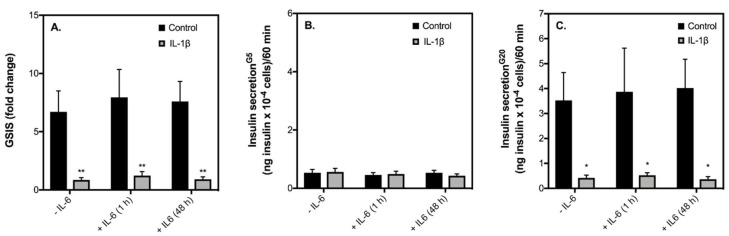
IL-1β-induced defects in insulin secretion are not prevented by IL-6. INS-1 832/3 cells were exposed for 48 h to 2 ng/mL IL-1β in RPMI with or without 10 pg/mL IL-6. For acute IL-6 effects, a separate set of cells pre-exposed to 48 h 2ng/mL IL-1β were treated with 10 pg/mL IL-6 for 1 h prior to insulin secretion assays. Control cells were incubated in the same media lacking IL-1β. IL-6 effects were determined on GSIS (**A**), basal (G5) insulin secretion (**B**), and high glucose (G20) insulin secretion (**C**). Data are means ± SEM of 4 independent experiments with each condition repeated 4–5 times. Mean differences were tested for statistical significance by 2-way ANOVA: asterisks indicate statistically significant differences from control cells without IL-1β (* *p* < 0.05 and ** *p* < 0.01).

**Figure 5:**
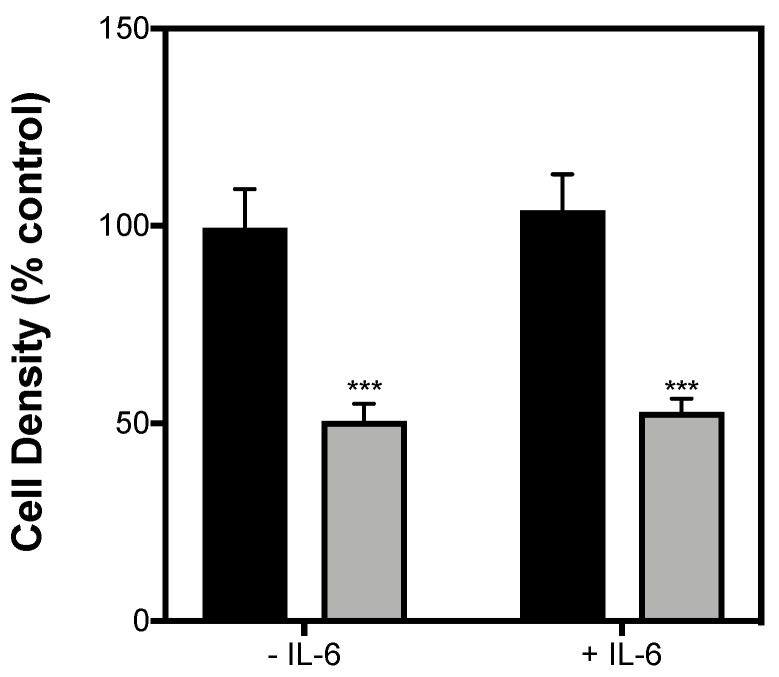
IL-1β-induced changes in cell density are not prevented by IL-6. INS-1 832/3 cells were exposed for 48 h to 2 ng/mL IL-1β in RPMI with or without 10 pg/mL IL-6 (grey bars). Control cells were incubated in the same media lacking IL-1β (black bars). IL-6 effects were determined on cell density as a percentage from equivalent control cells. Data are means ± SEM of 4 independent experiments with each condition repeated 4–5 times. Mean differences were tested for statistical significance by 2-way ANOVA: asterisks indicate statistically significant differences from control cells without IL-1β (*** *p* < 0.01).

In addition to elevated nonesterified fatty acids (NEFAs) and glucose, elevated levels of the proinflammatory cytokine IL-1β is associated with the pathophysiology of T2D [22]. Moreover, we [21] and others [13] have recently found that serum conditioned by exercise containing elevated levels of IL-6 impacts the viability of insulin-secreting cells and pancreatic islets exposed to proinflammatory cytokines. To examine if this is the case with β-cell function, we exposed INS-1 832/3 cells to IL-1β for 48 h in the presence and absence of exogenous IL-6 (10 pg/mL) and assessed acute insulin release. To account for acute IL-6 effects, we also treated INS-1 832/3 cells pre-exposed with IL-1β to IL-6 for 1 h. As expected, 48-h exposure to IL-1β blunts GSIS by INS-1 832/3 cells (Figure 4A). Since basal insulin release is unaffected by IL-1β (Figure 4B), this significant loss in GSIS is owing to a decreased insulin secretory response of cells to 20 mM glucose (Figure 4C). IL-1β had similar effects on insulin secretion in cells co-exposed with IL-6 for 48 h (Figure 4). Moreover, acute IL-6 neither restores nor worsens the loss of insulin secretion caused by IL-1β (Figure 4). Contrary to reports on cytokine-induced β-cell apoptosis [23,24], we also failed to provide persuasive support for a protective effect of IL-6 against IL-1β-induced changes in INS-1 832/3 cell density (Figure 5). 

### 2.3. Contraction-Mediated Increases in IL-6 from C2C12 Myotubes Has No Effect on Insulin Secretion by INS-1 832/3 Cells

**Figure 6:**
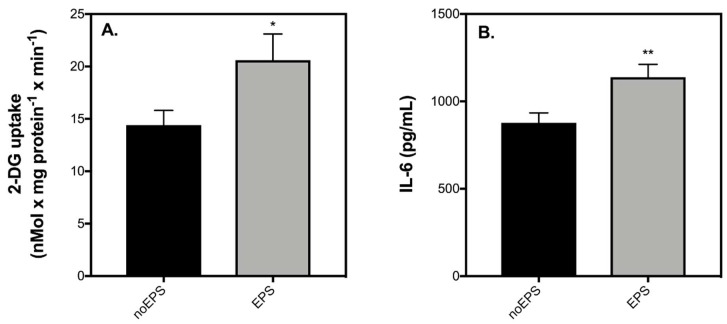
Electrical pulse stimulation stimulates glucose uptake in C2C12 myotubes and increases the secretion of IL-6. C2C12 myotubes were exposed with or without 24 h EPS. Effects of EPS were determined on glucose uptake (**A**) and IL-6 secretion (**B**). Data are means ± SEM of 4–9 independent experiments with each condition run in triplicate. Mean differences were tested for statistical significance by Paired *t*-test: asterisks indicate statistically significant differences from noEPS condition (* *p* < 0.05 and ** *p* < 0.01).

**Figure 7:**
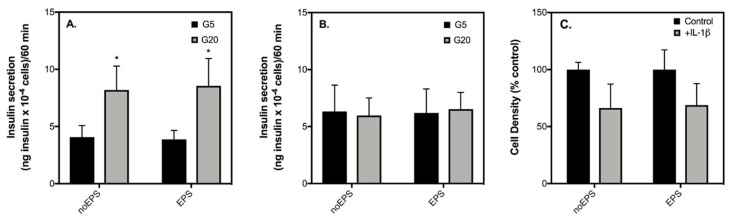
Conditioned media from “trained” skeletal muscle does not prevent IL-1β-induced decreases in insulin secretion. INS-1 832.3 cells were exposed with (**B**) or without (**A**) 24 h IL-1β combined with EPS or non-EPS-treated C2C12 muscle-cell conditioned medium. Effects of conditioned medium ± EPS were determined on basal (G5) insulin secretion (black bars—**A**,**B**), high glucose (G20) insulin secretion (grey bars—**A**,**B**), and cell density (**C**). Data are means ± SEM of 4 independent experiments with each condition repeated 4–5 times. Mean differences were tested for statistical significance by 2-way ANOVA: asterisks indicate statistically significant differences from basal glucose condition (* *p* < 0.05).

It has been suggested that IL-6 from skeletal muscle may act distinctly from IL-6 secreted during inflammation by immune cells or adipose tissue [25]. To account for this possibility, we also probed the effect of muscle-derived IL-6 on the insulin secretory function of healthy and IL-1β-exposed INS-1 832/3 cells by using muscle-conditioned medium (CM) from contracting C2C12 muscle myotubes. Replicating in vivo effects of exercise [21,26], stimulation of C2C12 myotubes with EPS significantly enhances glucose uptake (Figure 6A) and the secretion of IL-6 (Figure 6B). Consistent with previous observations shown in Figure 2, 20 mM glucose significantly enhances insulin secretion by INS-1 832/3 cells incubated with CM (Figure 7A). The effect of glucose on insulin secretion is similar regardless of EPS during the medium conditioning (Figure 7A). Coherent with the effects of IL-1β shown in Figure 4 and Figure 5, IL-1β has no effect on basal insulin release (Figure 7B), blunts insulin secretion in response to 20 mM glucose (Figure 7B), and lowers cell density (Figure 7C) of INS-1 832/3 cells exposed to CM. These IL-1β effects, notably, persist irrespectively of EPS during the medium conditioning (Figure 7). Our data suggest that a direct effect of IL-6 is unlikely responsible for the improvements in insulin secretion observed in patients with T2D following exercise and question the validity of IL-6 in mediating direct β-cell effects in health and disease.

## 3. Discussion

At physiological concentrations, IL-6 has no significant effect on insulin secretion by INS-1 832/3 cells (Figure 1). The finding that IL-6 at low pg/mL concentrations does not impair insulin secretion by normal INS-1 832/3 cells is of interest because it has recently come to light that IL-6 may have positive as well as negative effects in β-cells, especially at lower concentrations that mimic those that are shown to be present in circulation after exercise [18]. Given that IL-6 is secreted by skeletal muscle during exercise [27] and that both “exercise-conditioned serum” and “muscle-conditioned medium” containing elevated IL-6 impacts β-cell apoptosis in response to proinflammatory cytokines [13,21], we explored the effect of an exercise-relevant concentration of IL-6 on β-cell function under diabetic-like conditions.

### 3.1. IL-6 Effects on INS-1 832/3 Cells Exposed to Diabetic-Like Conditions

As expected from the typical behavior of native human β-cells, we show that in INS-1 832/3 cells, GSIS is significantly elevated in response to 20 mM glucose (Figure 2). Consistent with reports in BRIN-BD11 cells and isolated mouse islets [18], we show that acute IL-6 treatment at an exercise-relevant concentration of 10 pg/mL does not significantly affect GSIS or the amount of insulin released by INS-1 832/3 cells at 5 or 20 mM glucose (Figure 2). Confirming previous reports by us [28] and others [29], we show that diabetic-like glucotoxic and glucolipotoxic conditions lowers GSIS from INS-1 832/3 cells (Figure 3A,D) by lowering the amount of insulin secreted at a stimulatory glucose concentration (Figure 3C,F). Of interest, insulin secretory defects caused by diabetic-like glucotoxic and glucolipotoxic conditions (Figure 3) are not restored or exacerbated by acute pre-treatment of IL-6 (Figure 3). Collectively, these data suggest that a transient IL-6 response at an exercise-relevant concentration is unlikely associated with regulating insulin secretion from β-cells under healthy or T2D conditions associated with elevated glucose and/or NEFAs. 

In addition to elevated glucose and NEFAs, the pathophysiology of T2D has more recently been associated with increased inflammation through elevated levels of proinflammatory cytokines [30]. For example, pancreatic islets from T2D patients show increased expression levels of IL-1β. It is also well established that incubation of pancreatic islets [31] or cultured β-cell lines [32,33] to cytokines in vitro significantly impairs GSIS. Moreover, prolonged cytokine exposure leads to increased β-cell apoptosis [34] and is therefore linked with the loss of β-cell mass as well as their functional demise in T2D. In agreement with previous observations [33], we show that exposure of IL-1β for 48 h completely blunts GSIS from INS-1 832/3 cells (Figure 4A) by restricting insulin release at a stimulatory glucose concentration (Figure 4C). Notably, acute IL-6 treatment does not improve the loss of insulin secretory dysfunction caused by IL-1β (Figure 4). Given that it has been reported previously that prolonged IL-6 treatment protects against cytokine-induced apoptosis in cultured β-cells and islets [13], we also co-exposed INS-1 832/3 cells to IL-1β plus IL-6 for 48 h. Unlike the case with apoptosis, IL-6 was unable to prevent the loss of insulin secretory function caused by IL-1β under our conditions (Figure 4). Differing from reports on cytokine-induced β-cell apoptosis [23,24], we also failed to provide convincing evidence for the protective effect of IL-6 against IL-1β-induced cell loss (Figure 5).

### 3.2. Exercise-Induced IL-6 Effects against IL-1β-Induced INS-1 832/3 Cell Dysfunction

Despite failing to provide sufficient evidence for a protective role of IL-6 on the function or viability of INS-1 832/3 cells in our study, previous reports demonstrating a protective IL-6 effect against cytokine-induced β-cell apoptosis are convincing [8,13,14]. It is indeed possible that the reason for this discrepancy is related to the source of IL-6. For example, in the two studies that demonstrate a conclusive protective IL-6 effect against cytokine-induced apoptosis [13,14], IL-6 was administered to cells or islets in “exercise-conditioned serum” or “muscle-conditioned medium”. To account for this disparity, we exposed INS-1 832/3 cells to muscle-conditioned medium collected from C2C12 cells stimulated with or without EPS in the presence or absence of IL-1β for 24 h. To confirm our model of exercise using EPS, we show that both glucose uptake by C2C12 cells and secretion of IL-6 are significantly elevated following EPS (Figure 6). It should be noted, however, that although contraction mediates a significant increase in IL-6 representing physiology, the absolute concentration of IL-6 from C2C12 conditioned media is nearly 100-fold higher in CM than what would be expected under physiological conditions in humans. Moreover, CM contains many other myokines in addition to IL-6 which may impact the function or viability of β-cells [11]. That said, even with such high IL-6 concentrations, conditioned medium collected from contracting C2C12 myotubes neither improved nor worsened insulin secretory function of INS-1 832/3 cells exposed with (Figure 7B) or without (Figure 7A) IL-1β. We also show that in the presence of CM, IL-1β has similar effects on cell loss irrespective of whether the muscle cell medium was conditioned with EPS (Figure 7C). Collectively, our data suggest that an acute direct improvement in β-cell function by IL-6 is unlikely accountable for the exercise-induced benefit of β-cell function in patients with T2D [6,7]. 

It is worth noting that in our experiments, IL-6 was applied at a physiologically relevant post-exercise concentration [21,35,36] that is much lower than concentrations of IL-6 typically applied in vitro. This may explain discrepancies between our data and those published by others [8,23,37]. As our experiments are limited by the use of a single cultured β-cell line, we cannot at this stage exclude the possibility that IL-6 has functional effects on primary or other β-cell lines or that IL-6 indirectly mediates insulin secretion by β-cells, via GLP-1, for example, as shown in mice by Ellingsgaard et al. That being said, a role of IL-6 in mediating positive anti-inflammatory effects in β-cells has not to date been shown in humans and appears strange given that the action of IL-6 occurs via trans-signaling in isolated pancreatic islets which is classically related to a proinflammatory response [38]. Furthermore, plasma IL-6 levels are elevated in patients with metabolic diseases such as obesity and T2D [39], which are linked to hepatic cell dysfunction and consequent insulin resistance [40]. To further understand the role of muscle-derived IL-6 during exercise and its possible role in mediating insulin secretion in humans, further studies are required whereby acute insulin release of healthy and T2D human islets are examined following exposure to femoral human serum ± exercise in the presence and absence of IL-6. 

## 4. Materials and Methods

### 4.1. INS-1E 832/3 Cell Culture

Rat INS-1 832/3 insulinoma cells were maintained in RPMI-1640 medium (GIBCO #11879020) containing 11 mM glucose, 10% (*v*/*v*) fetal bovine serum (FBS), 1 mM sodium pyruvate, 50 U/mL penicillin, 10 mM HEPES, 50 μg/mL streptomycin, 2 mM GlutaMAX, and 500 μM β-mercaptoethanol. For experimental conditions, cells were seeded at 1 × 10^5^ cells/well in 96-well microplates with RPMI as described above but with 5 mM glucose. At 75–85% confluence, cells were treated with RPMI containing 5, 10, or 20 mM glucose ± BSA conjugated palmitate for 48 h, RPMI containing 5 mM glucose ± 2 ng/mL IL-1β with or without IL-6 (10 pg/mL) for 48 h, or C2C12 conditioned media ± EPS containing 5 mM glucose ± 2 ng/mL IL-1β for 24 h. Prior to experiments, INS-1 832/3 cells were washed into fresh RPMI medium containing 5 mM glucose ± IL-6 (at concentrations stated in figure legends) for 1 h unless otherwise stated. Palmitate was conjugated to bovine serum albumin (BSA) at a molar ratio of 7:1, yielding an estimated free fatty acid level of 20 nM, assuming binding parameters reported by Huber et al. (2006) [41]. FBS was omitted from growth medium for palmitate experiments and BSA was used as a vehicle control. The cytokines used in this study were from rat and purchased from R&D systems (Minneapolis, MN, USA). 

### 4.2. Insulin Secretion

INS-1 832/3 cells seeded and exposed to treatments as described above were washed into warm (37 °C) Krebs-Ringer HEPES (KRH) buffer comprising 135 mM NaCl, 3.6 mM KCl, 10 mM HEPES (pH 7.4), 0.5 mM MgCl_2_, 1.5 mM CaCl_2_, 0.5 mM NaH_2_PO_4_, 2 mM GlutaMAX, 5 mM glucose, and 0.2% (*w*/*v*) BSA. After 1 h, cells were washed into fresh KRH and incubated under air at 37 °C for 60 min with continuous shaking at 250 rpm. Next, supernatants were collected on ice and replaced with KRH containing 20 mM glucose. After another 60-min incubation, supernatants were collected on ice. Supernatants were assayed for insulin by homogenous time-resolved fluorescence (#62IN1PEG, Cisbio Bioassays, Codolet, France). Secreted insulin was normalised to cell density (see below).

### 4.3. Muscle-Conditioned Medium

Mouse skeletal muscle C2C12 myoblasts (CRL-1772) were maintained in Dulbeccos’s Modified Eagles Medium (DMEM: GIBCO #11966025) containing 5 mM glucose, 10% (*v*/*v*) fetal bovine serum (FBS), 1 mM sodium pyruvate, 100 U/mL penicillin, 100 μg/mL streptomycin, and 1 mM GlutaMAX. For differentiation to myotubes, cells were seeded in 6-well microplates at a density of 2 × 10^5^ cells/well and, at 85–95% confluence, washed into fresh DMEM containing 5 mM glucose, 2% (*v*/*v*) horse serum, 1 mM sodium pyruvate, 100 U/mL penicillin, 100 μg/mL streptomycin, and 1 mM GlutaMAX (differentiation medium). Differentiation medium was replaced daily until myotubes had formed. Visual inspection using an inverted light microscope indicated that complete differentiation took 5 days. Cell passages between 8 and 15 were used for experimentation. After 5 days of differentiation, myotubes were washed into fresh DMEM differentiation culture medium (described above) and treated for 24 h with or without electrical pulse stimulation (EPS: 11.5V, 1 Hz, using 2 milliseconds pulses; C-Pace EP, IonOptix, Westwood, CA, USA). In cells treated with EPS, muscle contraction was confirmed by visual inspection using an inverted light microscope. The EPS protocol used for our experiments is based on exercise training models developed in C2C12 cells [12] and mimics aspects of muscle contraction, for example, increased IL-6 secretion and enhanced 2-deoxyglucose glucose uptake. After 24 h, myotube-conditioned culture medium ± EPS was collected, filter-sterilised using 0.45 µM millipore syringe filters, and stored at −80 °C until used for crosstalk experiments with INS-1 832/3 cells.

### 4.4. Glucose Uptake

2-Deoxyglucose (2DG) uptake was measured as described previously by [42]. Briefly, C2C12 myotubes treated with or without EPS for 24 h (cf. 2.3) were washed and incubated in serum-free DMEM medium supplemented with 5 mM glucose, 1 mM sodium pyruvate, 100 U/mL penicillin, 100 μg/mL streptomycin, and 1 mM GlutaMAX for 2 h at 37 °C. Cells were then incubated for 20 min in the Krebs-Ringer bicarbonate buffer containing 115 mM NaCl, 4.7 mM KCl, 10 mM HEPES, 1.2 mM MgSO_4_, 1.28 mM CaCl_2_, 1.2 mM KH_2_PO_4_, 24 mM NaHCO_3_, 0.1% BSA, and 1 mM 2-DG. Subsequently, cells were washed twice with phosphate-buffered saline and lysed via agitated incubation in the presence of 0.1 N NaOH for 10 min at 65 °C and a further 50 min at 85 °C. Lysates were solubilised with 0.1 N HCl and then incubated, whilst shaking, for 10 min at room temperature in 200 mM triethanolamine, pH 8. Lysates were transferred to a 96-well plate containing assay medium with 50 mM triethanolamine (pH 8), 50 mM KCl, 15 U/mL glucose-6-phosphate dehydrogenase, 0.2 U/mL diphorase, 0.1 mM NADP, 0.02% (*w*/*v*) BSA, and 2 μM resazurin (Invitrogen, Carlsbad, CA, USA). This reaction mixture was incubated at 37 °C for 60 min to allow 2DG-dependent reduction of resazurin to the fluorescent resorufin (λ_ex/em_ = 540/590 nm), which was detected using a FLUOstar Omega plate reader (BMG Labtech, Offenburg, Germany). Glucose uptake was normalised to protein concentration as determined using the Bradford protein assay (Bio-Rad, Hercules, CA, USA). 

### 4.5. IL-6 Secretion

IL-6 was measured in conditioned medium collected from resting or contracting C2C12 skeletal muscle myotubes (cf. 2.3). Aliquots of conditioned media were assayed for secreted IL-6 by homogenous time-resolved fluorescence (#63ADK043PEB, Cisbio Bioassays, Codolet, France) using the manufacturer’s instructions. 

### 4.6. Cell Density

Density of attached cells was determined in 96-well plates by 4′,6-diamidino-2′-phenylindole dihydrochloride (DAPI) fluorescence [43]. Cells seeded and treated as described in the “insulin secretion” section were washed with phosphate-buffered saline (PBS) and fixed in 4% (*v*/*v*) paraformaldehyde (ThermoFisher Scientific (Waltham, MA, USA), #28908). After fixation, cells were washed again in PBS and then loaded with 0.5 μg/mL DAPI. To limit background detection after loading with DAPI, cells were washed 4 times with PBS. DAPI fluorescence (λ_ex/em_ = 350/460 nm) was detected using a FLUOstar omega plate reader (BMG Labtech, Offenburg, Germany ) in fluorescence intensity, bottom-reading, and well-scanning mode.

### 4.7. Statistical Analysis

Significance of mean differences was tested for by paired *t*-tests (Figure 6) and one-way (Figure 1) or two-way (all other figures) ANOVA applying Fisher’s LSD post hoc analysis using GraphPad Prism Version 7.0 for Mac OS X (GraphPad software, San Diego, CA, USA). Data are presented as means ± SEM.

## 5. Conclusions

Our data deepen the understanding of the link between IL-6 and glucose-stimulated insulin secretion in β-cells under healthy and diabetic-like conditions. We show that at an exercise-relevant concentration, IL-6 neither alleviates nor exacerbates β-cell failure and is therefore unlikely to mediate direct crosstalk effects between skeletal muscle and β-cells under diabetic-like conditions.

## Abbreviations

β-cell	Pancreatic beta-cell
T2D	Type 2 diabetes
TD1	Type 1 diabetes
GSIS	Glucose-stimulated insulin secretion
NEFA	Non-esterified fatty acids
KRH	Krebs-Ringer HEPES buffer
CM	Conditioned medium
BSA	Bovine serum albumin
HEPES	4-(2-hydroxyethyl)-1-piperazineethanesulfonic acid
DAPI	4′,6-diamidino-2′-phenylindole dihydrochloride
EPS	Electrical pulse stimulation
IL-1β	Interleukin-1 beta
IL-6	Interleukin-6
